# Editorial: Sonification, Perceptualizing Biological Information

**DOI:** 10.3389/fnins.2020.00550

**Published:** 2020-06-04

**Authors:** Diego Minciacchi, David Rosenboom, Riccardo Bravi, Erez James Cohen

**Affiliations:** ^1^Physiological Sciences Section, Department of Experimental and Clinical Medicine, University of Florence, Florence, Italy; ^2^The Herb Alpert School of Music, California Institute of the Arts, Valencia, CA, United States

**Keywords:** audio display, music, movement, sound, sonification

Sonification is a subtype of auditory displays that use sound structures devoid of linguistic elements to represent information. Kramer et al. (1999) have neatly defined sonification as “the transformation of data relations into perceived relations in an acoustic signal for the purposes of facilitating communication or interpretation.” The sense of hearing has the potential to convey in a simple way information that is complementary or alternative to visualization. Applications of sonification are by no means recent, in fact from early formulation of auditing, such as comparing the sound of commodities or “hearing of accounts” in Mesopotamia, as early as 3500 BCE (Worrall, 2009), to the three inventions from the nineteenth century, the Bell telephone, the Edison phonograph, and the Marconi radiotelegraphy, sound and audio were used and transformed to convey information. Many of these developments included translations of information into sound and were so powerful and rich of outcomes to change ultimately our relation to hearing in general. Commonly, implementations of sound constructions for information display are described for alarms, alerts, and warnings, status, process, and monitoring messages, data exploration, and finally for entertainment, sports, exercise, and art (Walker and Nees, 2011).

Though simplistic forms of sonification were always employed to represent phenomena from the physical world, the rapid developments at the end of last century in psychoacoustics, data manipulation, sound synthesis, and sonification techniques, caused an outburst of research in this field (Kramer, 1994; Hermann et al., 2011). With technological advancements the amount of data being processed is immense. In current research, regardless of the field, resorting to datamining is often inevitable. For this exact reason, representation of data has an important role. For certain applications, the most immediate representation of data is confined to the visual. However, there are still limitations when it comes to the visual representation of data by itself. For example, within medical diagnostics, even the most refined machinery is limited by our own perception of reality. Also, a visual representation is often a static one, and therefore a partial representation. Audio on the other hand, a time based experience by definition, is a dynamic representation, though also limited by our perception, it does add another dimension to our exploration of data. The addition of another dimension for evaluating data therefore, appears not only to be necessary but also natural. Any interaction of this world is done with our senses, and as such the more participating senses are during the exploration, the clearer the image and the subtleties of the world. Conversely, confinement of the exploration to a single dimensionality, is bound to limit our understanding.

With these premises, the implementation of sonification into various fields could provide major advances for the interpretation of data. Specifically, the approach was recently integrated within the field of neuroscience to facilitate the understanding of biological mechanisms and structures. Applications are manifold including behavioral monitoring, complex data extracting and analysis, algorithm development and interface implementations. Also, central application areas such as data navigation, status and process monitoring, motor tutoring for sports and exercise, and assistive technology for people with disabilities, became sites of strategic interest for sonification. Finally, sonification is increasingly being used as an aesthetic concept and method in the artistic and entertainment domain.

The articles presented in this Research Topic can be divided into three general sections. The first section includes articles concentrated on the implementations of sonification in movement research. Specifically, studies were oriented toward the examination of the effect of augmented sensorial feedback, by means of sonification, on motor learning. Movement sonification is applied on motor learning in sports (Effenberg et al.) and to test augmented sonified feedback on the motor learning of a novel joint coordination pattern (Fujii et al.). This line was further expanded by examining the possible benefits of sonification methods for sensori-motor learning with movement sonification (Bevilacqua et al.) and a perception-action approach to sonification used as feedback for skill learning (Dyer et al.), which may lead researchers toward encouraging applications in rehabilitation, sport training or product design. The section is concluding with interactive and real time implementations of sonification methods to the benefit of psychological and physical optimization of sports and motor rehabilitation tasks through the main functions Motivate, Monitor, and Modify of the 3Mo model (Maes et al.) and to analyze children's spontaneous movement in terms of energy, smoothness and directness (Frid et al.).

The second section focuses mainly on sonification as an open-ended design task to construct general sound information processes that translate data into sound maintaining reproducibility when sources exhibit non-linear properties of self-organization and emergent behavior (Choi) and a specific supportive auditory tool to aid in diagnosing patients with different levels of Alzheimer's that introduces an audible parameter mapped upon different brain's lobes (Gionfrida and Roginska), and an auditory interface for intuitive detection and management of anxiety from physiological signals (Cheung et al.).

The third section overviews implementations of sonification methods as means of therapeutics. For rehabilitation purposes in socially relevant movement disorders such as Parkinson's disease, where sonification has been used to help relearn gait movements and to reduce freezing episodes (Mezzarobba et al.; Murgia et al.), and stroke patients, where is presented an innovative musical sonification therapy, designed to retrain patients' gross motor functions of the upper extremity (Scholz et al.). Furthermore, a real-time auditory feedback based on movement sonification approach is used to compensate proprioceptively deafferented subjects (Danna and Velay), and a modification of the environmental sound training procedure is utilized to enhance neural plasticity, and reconstruct auditory representations that have become degraded after chronic use of cochlear implants (Altieri et al.).

Though the benefits of sonification are clear and become well-evidenced in this topic and most recent literature (see, Schaffert et al., 2019), widespread implementation is still scarce. We believe that a collective endeavor is necessary to put theory into practice and, perhaps, to start to exploit the large potential of sonifications.

## Author Contributions

All authors listed have made a substantial, direct and intellectual contribution to the work, and approved it for publication.

## Conflict of Interest

The authors declare that the research was conducted in the absence of any commercial or financial relationships that could be construed as a potential conflict of interest.

## References

[B1] Hermann T. Hunt A. Neuhoff J. (eds.). (2011). The Sonification Handbook. Berlin: Logos Verlag Berlin GmbH.

[B2] Kramer G. (ed.). (1994). Auditory Display: Sonification, Audification, and Auditory Interfaces. Boca Raton, FL: CRC Press.

[B3] Kramer G. Walker B. Bonebright T. Cook P. Flowers J. H. Miner N. (1999). Sonification Report: Status of the Field and Research Agenda. Faculty Publications, Department of Psychology.

[B4] Schaffert N. Janzen T. B. Mattes K. Thaut M. H. (2019). A review on the relationship between sound and movement in sports and rehabilitation. Front. Psychol. 10:244. 10.3389/fpsyg.2019.00244 30809175 PMC6379478

[B5] Walker B. Nees M. (2011). “Theory of sonification,” in The Sonification Handbook, eds Hermann T. Hunt A. Neuhoff J. (Berlin: Logos Publishing House, 9–39.

[B6] Worrall D. (2009). Sonification and information: concepts, instruments and techniques (Doctoral thesis). University of Camberra (AU).

